# 589 Burn Center Trainees: Not Just for Surgery or Plastic Surgery Residents

**DOI:** 10.1093/jbcr/irac012.218

**Published:** 2022-03-23

**Authors:** Lauren B Nosanov, Abraham Houng

**Affiliations:** New York Presbyterian / Weill Cornell Medical Center, New York, New York; Weill Cornell Medical College, New York, New York

## Abstract

**Introduction:**

The Burn unit offers a unique training environment for residents. The Accreditation Council for Graduate Medical Education (ACGME) requires all General Surgery trainees to have knowledge of burn physiology and experience with initial burn management. However, there are no burn requirements for other ACGME-sponsored training programs except for Plastic Surgery. Since care of the burned patient spans multiple settings – the intensive care unit, operating room, wards, outpatient clinic, and the emergency department – having residents from varied specialties might benefit not only the trainee, but also the Burn Center.

**Methods:**

A retrospective review of all residents rotated to the burn center of an American Burn Association verified unit was performed. Data from the 7/2018-6/2018 academic year were collected by analyzing resident rotational and call schedules of both intra institutional and inter-institutional residents. The specific time period was chosen to account for COVID affecting the number of residents more recently.

**Results:**

A total of 48 residents rotated at the burn center during the studied academic year. Within the institution, there were 34 residents (71%): 12 general surgery interns (8 categorical, 4 preliminary), 2 plastic surgery interns, 10 emergency medicine (EM) residents, and 10 anesthesia residents. There were 14 residents (29%) from 3 outside institutions: 3 plastic surgery residents, 8 surgery interns from one program, and 3 surgery interns from another program. All surgical specialty trainees were interns, whereas other specialties, EM and anesthesia, were PGY2 trainees.

**Conclusions:**

While most residents were from general surgery and plastic surgery programs (58%) due to ACGME requirement, a significant portion of the resident complement (42%) was from non-surgical specialties. EM residents gain competency in wound reading as well as burn critical care. Anesthesia residents learn surgical management of the burn patient and critical care procedures. Since the burn center is a tertiary referral center, having outside residents rotating in the burn unit might facilitate transfers and increase knowledge of proper resuscitation.

